# Toward an Aptamer-Based Biosensor for Cross-Serotype Detection of Botulinum Neurotoxin: Binding Mechanism from a Comprehensive Computational Investigation

**DOI:** 10.1021/acsomega.6c06201

**Published:** 2026-08-19

**Authors:** Ly Hai Nguyen, Lien Thi Ngoc Truong, Phi Long Nguyen, Quynh Luu-Manh, Yen Bao Pham, Thi Viet Bac Phung, Toan The Nguyen

**Affiliations:** † Institute of Theoretical and Applied Research, 374802 Duy Tan University, 1 Phung Chi Kien, Cau Giay, Hanoi 11300, Vietnam; ‡ School of Engineering and Technology, Duy Tan University, Da Nang 50000, Vietnam; § Vietnam - Korea Institute of Science and Technology, Hoa Lac High-Tech Park, km29 Thang Long Boulevard, Hanoi 13100, Vietnam; ∥ Center for Environmental Intelligence and College of Engineering & Computer Science, 561287 VinUniversity, Gia Lam, Hanoi 12400, Vietnam; ⊥ Faculty of Physics, 308298 University of Science, Vietnam National University - Hanoi, 334 Nguyen Trai, Thanh Xuan, Hanoi 11400, Vietnam; # Key Laboratory of Enzyme and Protein Technology, University of Science, Vietnam National University - Hanoi, 334 Nguyen Trai, Thanh Xuan, Hanoi 11400, Vietnam; ∇ Key Laboratory for Multiscale Simulation of Complex Systems, University of Science, Vietnam National University - Hanoi, 334 Nguyen Trai, Thanh Xuan, Hanoi 11400, Vietnam

## Abstract

Botulism is a potentially fatal illness in humans and many animals caused by the botulinum neurotoxins (BoNTs) produced mainly by *Clostridium botulinum*, which can be found in a wide variety of foods. The treatment of this illness requires antitoxin drugs given to the patients within a 48 h window to be effective. Therefore, there is a strong need for the development of rapid and sensitive biosensors for the early detection of the toxins in foods. In this work, by using comprehensive atomistic computer modeling and simulation, we investigate aptamers that bind BoNT serotypes A, B, and C to serve as recognition elements in the biosensor design for cross-serotype detection. Known aptamers targeting BoNT/A and BoNT/C are investigated in this work to understand whether they can cross-react with other serotypes. Our results highlight the importance of structural relaxation of the aptamers, which the standard docking procedure cannot adequately capture. During molecular dynamics, most of the aptamer structural relaxation correlates with binding to the toxins. Among the aptamers chosen, we found two aptamers A4 and A5 that bind equally strongly to all serotypes. C5 binds equally strongly to its designated target of serotype C, but it binds significantly weaker to other serotypes. These most promising aptamers, based on the computational analyses, are all ssDNA aptamers and share a common feature of a DNA double helix of 5–6 nucleotide base pairs. For all aptamers investigated, there are two binding sites for an aptamer on BoNT, which are located on opposite sides of the protein. This suggests an experimental setup of a sandwich array to allow aptamers to bind to both binding sites for optimal sensitivities. Our computational results provide molecular mechanisms of binding and assist in further optimization and design of new aptamers for these botulinum neurotoxins.

## Graphical abstract

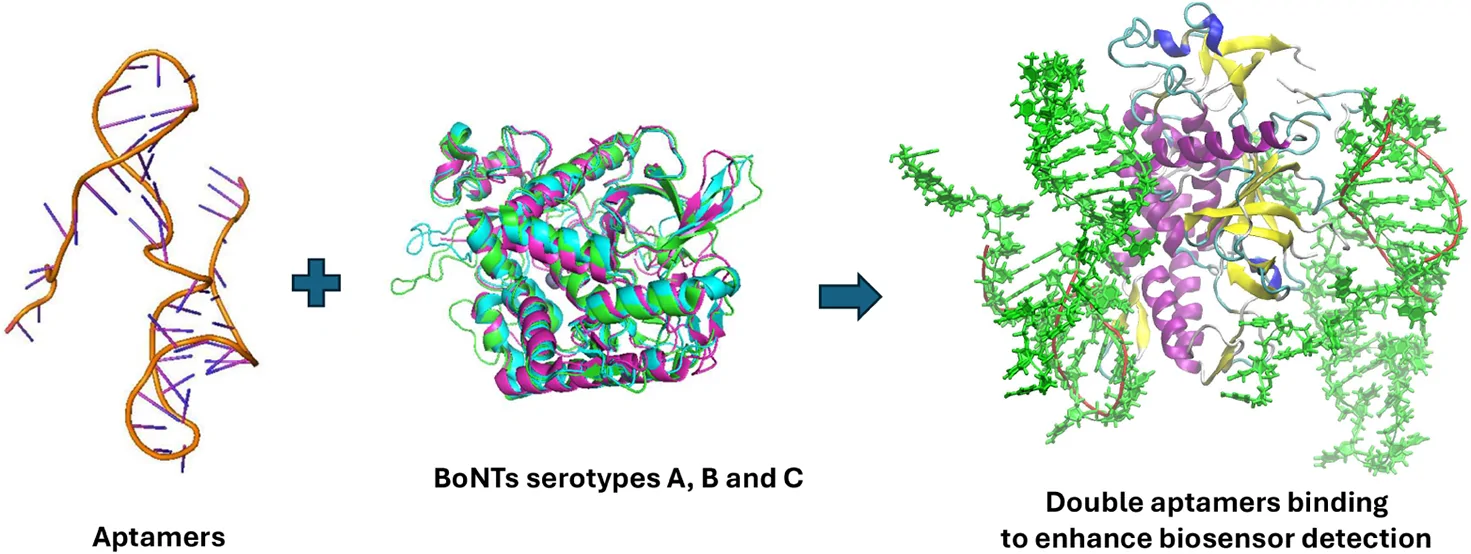

## 1 Introduction

Botulinum neurotoxin is recognized as one of the most potent toxins, capable of inducing fatal outcomes at concentrations as minimal as 1–3 ng/kg in human subjects.^1^ The bacterium *Clostridium botulinum*, a rod-shaped bacterium, produces botulinum neurotoxins that elicit neurological manifestations akin to those observed in various medical conditions such as stroke and meningitis. In addition to its detrimental effects on human health, research has demonstrated that at sublethal concentrations, botulinum neurotoxin has been effectively employed in the treatment of facial wrinkles, a utilization endorsed by the Food and Drug Administration (FDA).^2–4^

BoNT is categorized into seven different serotypes (A-G).^5^ The identification of an eighth serotype, designated as BoNT/H, has also been reported, which is a hybrid of serotypes A and F,^6^ BoNT serotypes C and D exhibit toxicity toward animals and avian species,^7^ especially botulism by BoNT/C has become an emerging and serious problem worldwide,^8^ notably in livestock, where it causes high mortality rates and substantial economic losses. BoNT/A, -B, -E, and -F are known to affect humans with serotype A recognized as the most potent neurotoxin, possessing a lethal dose estimated at 1 ng/kg. Additionally, BoNT/C has the potential to adversely impact human health as well.^9^ Each serotype of botulinum neurotoxin targets distinct physiological substrates, yet they share a unified mechanism of action. They bind specifically and with high affinity to active nerve synapses, internalized by endocytosis, and then, the enzymatically active protease subunit is translocated into the cytoplasm of the target cell.^10^ Collectively, these actions account for the pronounced potency and specificity exhibited by botulinum neurotoxins toward active neuromuscular junctions^11,12^ wherein these toxins inhibit the release of acetylcholine, a critical neurotransmitter. This inhibition prevents nerve signals from reaching the muscles, resulting in a temporary muscle paralysis. In therapeutic applications, this effect is used to treat conditions associated with muscle overactivity.

Botulinum neurotoxins are complex molecules composed of three domains as shown in Figure 1.^13–16^ The N-terminal light chain (LC) comprises the enzymatic domain, which functions as a zinc metalloprotease and is constituted of two independent functional domains: the translocation domain (HCT) and the receptor-binding domain (HCR). These three domains are structurally independent.^16^ Earlier studies showed that a C. botulinum-derived endopeptidase was responsible for nicking BoNT/A to generate the dichain toxin.^17–20^ The cleavage removed 10 amino acids at the junction between the LC and the HC, yielding a light chain of ≈438 amino acids, which is considered the active form of the LC.^21^ A reduction in the toxicity of several derivatives of BoNTs produced by *C. botulinum* in the unnicked form shows the importance of nicking. Due to this mechanism, the light chain of BoNT is usually the target to develop biosensors for detecting these neurotoxins.

**Figure 1:**
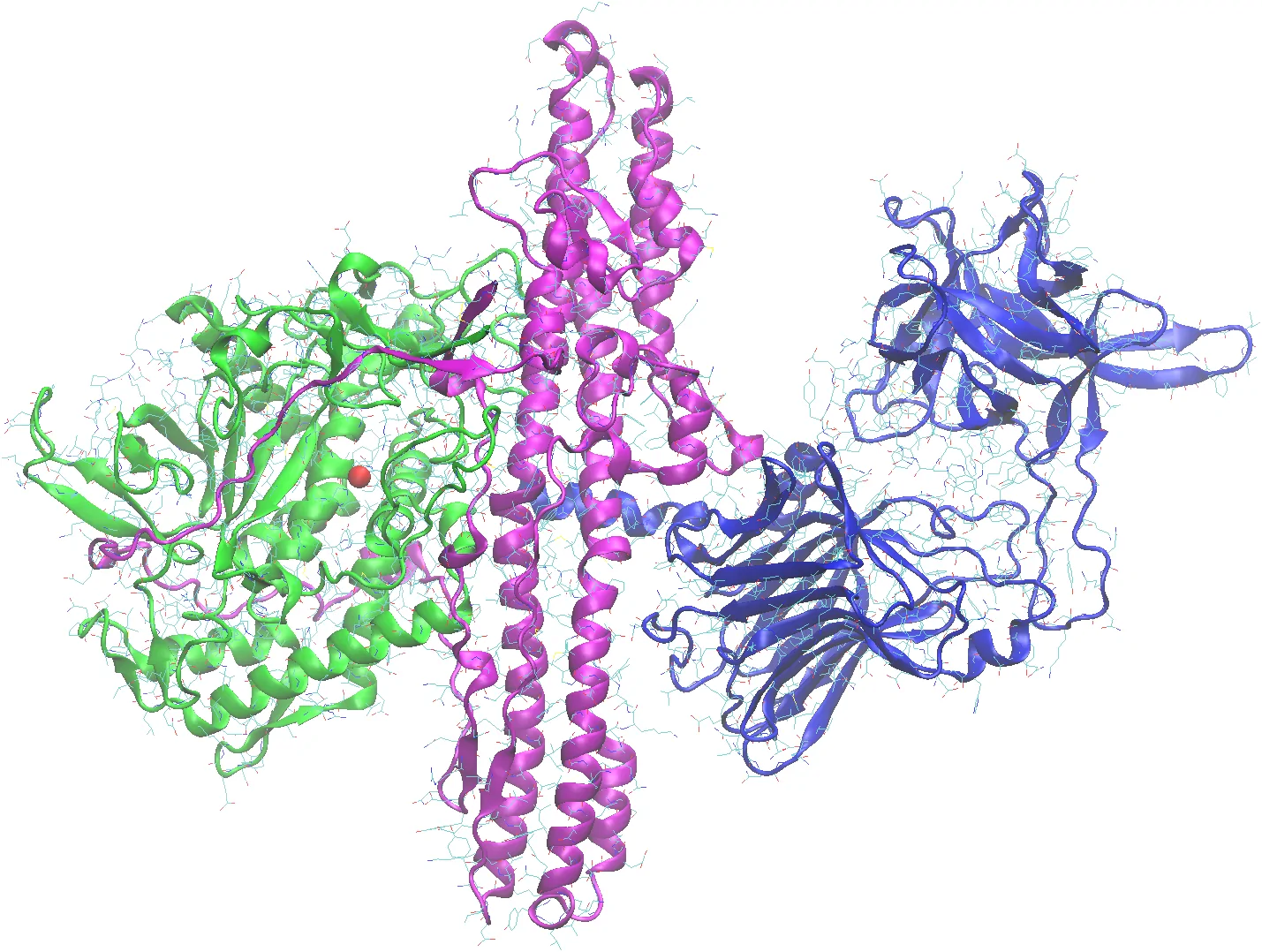
Botulinum neurotoxin protein consists of three domains: the catalytic domain, also called the light chain (LC), is shown in green color with zinc ion shown in red color; the heavy chain (HC) contains the translocator domain is shown in violet color and the receptor binding domain shown in blue color. Aptamers for biosensors for the detection of BoNT are designed to target the catalytic domain. Here, the BoNT/B three-dimensional structure at 1.90 Å resolution, obtained experimentally using the X–ray diffraction technique^13^ (PDB ID: 1EPW) is shown.

Conventional bioassays used for BoNT detection are expensive and time-consuming.^22^ Therefore, there is an urgent need to develop techniques that are rapid, specific, and reliable for the detection of botulism. For this purpose, aptamer-based biosensors present advantageous solutions. Aptamers are a type of single-stranded oligonucleotide, including RNA or single-stranded DNA, discovered in 1990.^23^ They exhibit diverse structures and shapes, such as helices, hairpins, loops, cloverleafs, and pseudoknots. Aptamers function and operate similarly to antibodies but offer advantages due to their stable three-dimensional structures, unaffected by environmental factors. They can be synthesized in large quantities at a low cost, and they have a long shelf life with limited degradation once modified, e.g., with 2′-OMe or 2′-F.^24^ In light of these benefits, aptamers are employed in a multitude of fields, including medical diagnostics, therapeutic interventions, cosmetic applications, and beyond.

In the present study, we employ physics-based computational simulations and analytical techniques to thoroughly elucidate the binding mechanisms of aptamers to botulinum neurotoxin (BoNT). In alignment with recent experimental trends,^25–28^ our objective is directed toward the detection of cross-serotype botulinum neurotoxin. It has been established that aptamers specifically engineered for BoNT/A also demonstrate effective binding to the BoNT serotypes B and E. Consequently, for the purpose of this cross-species investigation, established aptamer sequences that target BoNT/A and BoNT/C were selected for molecular-level computational analysis. By examining the structural and energetic characteristics of the aptamer-BoNT complexes and comparing features of various aptamers and serotypes, we identified several important characteristics of the most promising aptamer sequences for BoNT binding. Our findings contribute to the experimental selection or optimization of aptamers intended for biosensor applications in the detection of botulism.

## 2 Materials and Methods

### 2.1 Preparations of Ligands

From the experiment, we identified five superior candidate aptamers for each of the two target botulinum neurotoxins (BoNTs).^29,30^ These selected aptamers are enumerated in Table 1, which includes their primary sequences, the count of nucleotide bases, as well as their corresponding target BoNT. The abbreviated designations A1-A5 and C1–C5 are employed throughout this study to facilitate the identification of individual aptamers.

**Table 1:** List of Aptamers Selected for Investigation in This Work Are Selected from a List of Experimental Aptamers Known to Bind Well to Their Respective Target Protein

Aptamer	Residue sequence	Number of residues	Targeted BoNT serotype
A1	GACAGCGUGCCUAGAAGUCCAAGCUUAAAU	47	Type A
	AACCACGCUCGACAAGC		
A2	ACAACCCGGAACAACGUCUAACAGUGUACC	45	Type A
	AUAACCCGGCAUUCA		
A3	AUUCGGGCCCAGGAACCAACUAUAUAAAUG	47	Type A
	UCCCGAAUGCUUCGACG		
A4	CTATAGGGTACCCACTCAGGTACGCAGCTT	49	Type A
	TCTAGAATTAAGCTTAGGC		
A5	CATACGTTCGACTGCTACCCTCCACTTTTG	81	Type A
	ACGGCTTCCTCGGGATTATACGGCTAACCG		
	AGGGTGAGATGTACAGACTAG		
C1	GCAAAGGTTTTGAATAACCGCTGAGCTCTC	40	Type C
	CCGTTCTGCG		
C2	UGGUGGUCAGUGAGUGGUCGGGUCUCUGCA	40	Type C
	UCUCUGUGUG		
C3	GGAUCUGCUGUGUACCUCGCUGCUCGACUG	40	Type C
	GACUAGUCCG		
C4	CGGATGCGGGTAATTGAATTAGTACGGTTT	40	Type C
	GCCCCTTGTG		
C5	GGATCTGCTGTGTACCTCGCTGCTCGACTG	40	Type C
	GACTAGTCCG		

Starting from these primary sequences, the mFold Web server^31^ is employed to create the secondary structure of the aptamers. Subsequently, the RNAComposer Web server^32^ is used to create the three-dimensional structure. If the aptamer is an ssDNA molecule, we follow the recipe of refs 33,34 (see also ref 35 for a more comprehensive review of available tools) to construct their three-dimensional structure. An in-house code based on the Open Babel Python library is used to modify the backbone from ribose sugar to deoxyribose sugar and to replace the uracil base by the thymine base. The modified structures are subsequently subjected to energetic relaxation employing the steepest descent method with the MMFF94 force field to obtain an optimized structure.

It should be mentioned that our standard procedural approach for constructing atomistic models of aptamers using the mFold and RNAComposer tools does not facilitate the prediction of noncanonical motifs, such as G-quadruplexes or triplexes. Nevertheless, we have scanned our sequences utilizing the DNA Analyzer web portal^36^ and found no such noncanonical motifs. Consequently, our aptamer models are suitable for this purpose.

### 2.2 Docking Aptamer–BoNT Protein and Molecular Dynamics Simulation

The three-dimensional conformations of the light chain (LC) domain of the botulinum neurotoxin for serotypes A, B, and C have been downloaded from the Protein Data Bank, with the corresponding PDB ID codes being 3QIX for type A,^37^ 1F82 for type B,^38^ and 2QN0 for type C.^39^ The docking procedure of aptamers to botulinum neurotoxin is done using HADDOCK^40^ software using flexible docking, which allows for the molecules to have some small, limited degrees of local relaxation.

Given the inherent flexibility and substantial size of aptamers, the standard docking method using static structures cannot capture the non-negligible structural relaxation of the protein–aptamer complex upon binding. Consequently, we selected the most promising aptamers from the docking outcomes to conduct molecular dynamics (MD) simulations, aimed at elucidating their structural relaxations and interactions after the initial docked formation of the complex. In this work, for the purpose of cross-serotype detections, aptamers with the strongest docking score across all three serotypes are selected. For MD simulation, the GROMACS 2020.7 software package is used.^41^ The Amber99SB-ILDN force field^42^ is used to parametrize both the protein and the aptamer, ensuring maximal compatibility. The TIP3P model is used to parametrize water molecules. NaCl salt is added to the systems at a 150 mM concentration. The zinc-finger structure of the BoNT LC proteins is specifically taken care of as described in ref 43. The long-range electrostatic interaction is computed employing the Particle Mesh Ewald method.^44^ A cutoff radius of 1.2 nm is used for both electrostatic and van der Waals interactions.

The dimensions of the simulation box were selected to ensure that the complexes in neighboring periodic boxes were separated by a minimum distance of 3 nm. Considering that the electrostatic screening length at a 150 mM NaCl concentration is approximately 7 Å, this 3 nm separation is sufficiently extensive to mitigate the finite size effects arising from long-range electrostatic interactions among proteins in neighboring simulation boxes. In addition, this size of the simulation box is small enough to get results from MD simulations in a reasonable time regarding our current computational resources.

These systems first underwent optimization via the steepest descent method to avoid potential high-energy contacts of overlapping atoms after the initialization step. Subsequently, the systems were equilibrated at a 300 K temperature and 1 atm pressure using the V-rescale thermostat and the Berendsen barostat, respectively. During the equilibration phase using the NPT ensemble, restraints of 1000 kJ/mol were applied on heavy atoms of the protein and the aptamer, along all three coordinates. The restraints were then released for the production run simulation of a minimum of 200 ns, in order to perform statistical analysis of the systems. During the production run, Parrinello–Rahman barostat and velocity rescale thermostat are employed. The LINCS constraints on all bonds involving hydrogen atoms were used, thereby permitting a time step of 2 fs for the simulation.

### 2.3 Free Binding Energy and Other Analyses of Simulation Results

Snapshots of the system during the simulation were acquired at intervals of 100 ps. The VMD program^45^ is used for the purposes of visualization, spatial analysis, surface characterization, figure generation, and examination of molecular interactions. Certain analyses pertaining to the collective degrees of freedom of the molecules are conducted utilizing the open-source, community-driven PLUMED library,^46^ version 2.x,^47^ in conjunction with the GROMACS simulation package. For taking statistics in equilibrium, the first 50 ns of the simulation data is excluded from consideration. The molecular mechanics/generalized Born surface area (MM/GBSA) method is used for the estimation of binding free energy. The entropy contribution is omitted in the calculation, and only interaction energies are calculated. The MM/GBSA calculation method is widely recognized for efficiently balancing accuracy and computational efficiency. This is particularly significant in the context of simulation of large biological systems, where alchemical methods such as free energy perturbation or thermodynamic integration methods are extremely computationally costly and can be noisy. The gmxMMPBSA Python library^48^ is utilized for the actual computations of the binding free energy.

## 3 Results and Discussion

### 3.1 Docking of Aptamers to Botulinum Neurotoxin of Different Serotypes

The results of docking various aptamers to the three serotypes of botulinum neurotoxin A, B, and C are presented in Table 2. For comparative analysis, the two docking poses with the lowest docking free energies are listed for each system. The rationale for presenting two poses will be clear in the next subsection (see also Figure 2).

**Table 2:** Free Energies of Docking of 10 Aptamers Chosen to the Three BoNT Serotypes^a^

Aptamer	Type, #nu	Docking Pose	BoNT/A	BoNT/B	BoNT/C
A1	RNA, 47nu	1	–22.375	–25.620	–24.326
		2	–22.079	–23.764	–23.732
A2	RNA, 45nu	1	–28.759	–26.517	–22.443
		2	–24.670	–25.920	–22.223
A3	RNA, 47nu	1	–23.304	–23.757	–25.827
		2	–22.522	–22.550	–22.180
A4	DNA, 49nu	1	–29.510	–31.322	–30.027
		2	–28.902	–30.644	–29.035
A5	DNA, 81nu	1	–31.475	–35.229	–33.023
		2	–30.607	–34.801	–31.701
C1	DNA, 40nu	1	–33.252	–29.904	–28.548
		2	–31.093	–29.596	–27.596
C2	RNA, 40nu	1	–25.387	–25.337	–24.678
		2	–25.089	–23.807	–23.657
C3	DNA, 40nu	1	–23.481	–24.778	–28.563
		2	–22.791	–24.641	–23.997
C4	DNA, 40nu	1	–37.974	–34.236	–32.618
		2	–31.245	–33.800	–32.100
C5	DNA, 40nu	1	–31.872	–35.482	–33.248
		2	–30.413	–33.417	–29.343

a All values are in units of kcal/mol. For each system, the two lowest free energies are shown.

**Figure 2:**
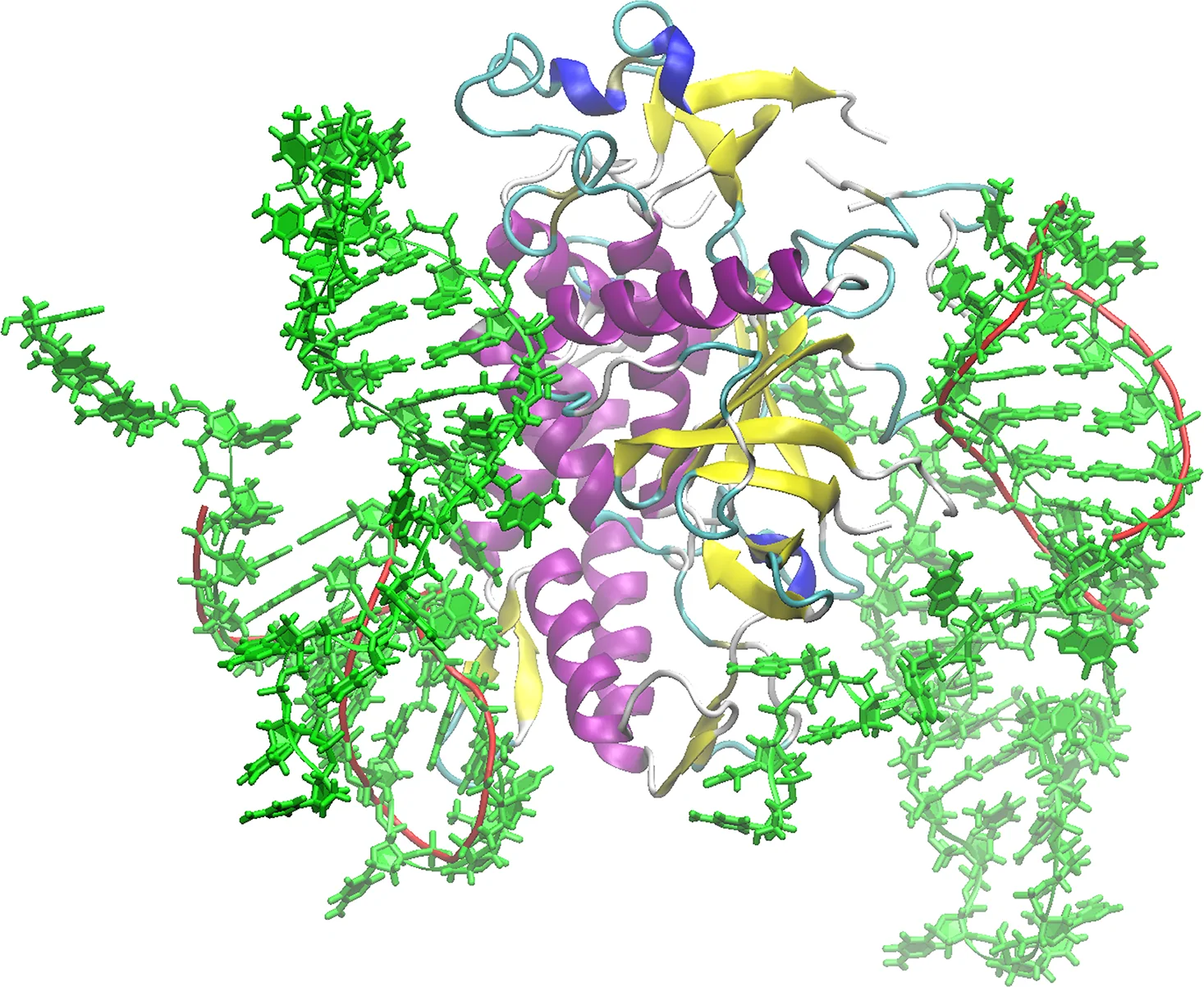
Two docking poses with the lowest free energies of binding for complexation of the aptamer A4 (shown in green colors) to the BoNT serotype A (shown at the center). The two poses are on opposite sides of the protein and do not overlap. The red tube is the hairpin structure of the aptamer that participates in binding. See the text for more discussion. Experimentally, one can utilize both binding sites to increase biosensor detection sensitively using a sandwich array.

All aptamers exhibit a binding free energy of −20 kcal/mol or stronger, indicating a robust affinity for all three variants of BoNT. This outcome is somewhat anticipated, as these aptamers have previously been demonstrated experimentally to be proficient candidates for binding to their corresponding target proteins. Specifically, these binding free energies translate into sub-nM binding affinity, somewhat stronger than available experimental data, ranging from 10 nM to 0.7 nM binding affinity.^29,30^ Nevertheless, they are acceptable for our purpose of comparative analyses. Importantly, the docking results reveal that these aptamers exhibit significant cross-binding capabilities with the other target proteins. The aptamer designated for BoNT/A demonstrates substantial affinity for the BoNT/B and BoNT/C targets, and conversely. This is also understandable since the three protein serotypes have very similar enzymatic functions; thus, despite differences in primary sequence, their structures share many similarities, not only the substrate-binding zinc-finger structures, but other domain structures as well.

Among the aptamers, however, aptamers A4, A5, C4, and C5 appear to have the most substantial binding free energies across all three serotypes. These aptamers were selected for subsequent molecular dynamics studies to elucidate the underlying physical mechanisms involved. Each complex will undergo simulation for a duration of at least 200 ns, which is sufficient for our objectives concerning further structural optimization and examination of the individual interactions between the aptamer and the protein.

### 3.2 Binding of an Aptamer to a Botulinum Neurotoxin

Before going into the comparison and discussion of different aptamers, their similarities, and differences, let us start by investigating the mechanism and the physicochemical properties of the binding of an aptamer to a botulinum neurotoxin. This helps to establish the basic physical concepts and terminology that will be used for later discussions. For a typical system, the aptamer A4 bound to its designated target BoNT type A is described in detail in this section. Although this aptamer is chosen because it is the first system that we simulate, the discussions presented in this section are equally applicable to elucidating the binding dynamics of other aptamer-BoNT complexes.

In Figure 2, the two top binding poses of this A4 aptamer to BoNT type A LC protein are shown. It is notably intriguing to observe that the two binding sites for this aptamer are on the opposite side of the protein with comparable binding free energies and no overlap. This implies that one can utilize a sandwich assay based on a pair of these aptamers to enhance the sensitivity of biosensors since each of them can bind to different sites. Of course, one should stress that this is only a modeling-based suggestion and not a confirmed fact for these BoNT serotypes. Nevertheless, this strategy indeed has been realized experimentally for BoNT/C serotype in ref 29. It is reasonable to expect that this strategy works equally well for other serotypes since they are all very similar in structure. Note that in both cases, the second hairpin of the aptamer (shown as red tubes in the same figure) participates significantly in complexation with the BoNT protein. As we will see later, this hairpin is a common feature of many of the most promising aptamers investigated in this work and may be instrumental in the design of aptamers.

To examine the structural stability of the binding conformation derived from molecular docking, as well as to analyze the dynamic relaxation of the aptamer and BoNT complex upon binding, a 200 ns molecular dynamics simulation of the optimal pose was conducted, as delineated in the Materials and Methods section. First, let us investigate the relaxation time of the system. Figure 3 shows the root-mean-square deviation (RMSD) of the protein and aptamer from their initial configurations as a function of time. Figure 3a shows the RMSD of the protein with respect to its starting configuration, while Figure 3b shows the RMSD of the aptamer with respect to its starting configuration. Both Figure 3a and b show that the RMSD values saturate at a later time, suggesting that our system has reached equilibrium. The protein is very stable, reaching equilibrium after only about 20 ns. The deviation from the starting point is also very small, of only 1.2 Å, much smaller than the typical value of 5 Å one usually observes for free protein in an aqueous solution. The binding of the BoNT/A protein with the aptamer has a stabilizing effect on the protein. In contrast, the aptamer demonstrates a longer duration to achieve equilibrium, approximately 50 ns. This is to be expected because the starting configuration was obtained from a static molecular docking approach, for which no relaxation or only very minimal local relaxation is allowed. Only through molecular dynamics simulation can the aptamer molecules explore more extensive configurations and adjust to optimal arrangements. Since the aptamer is a very flexible and extended single-stranded DNA molecule, the RMSD to reach equilibrium is much higher than that of the protein, of the order of 12 Å. Based on this RMSD analysis, we will exclude the first 50 ns from subsequent analyses to ensure that the statistical evaluations are performed under equilibrium conditions.

**Figure 3:**
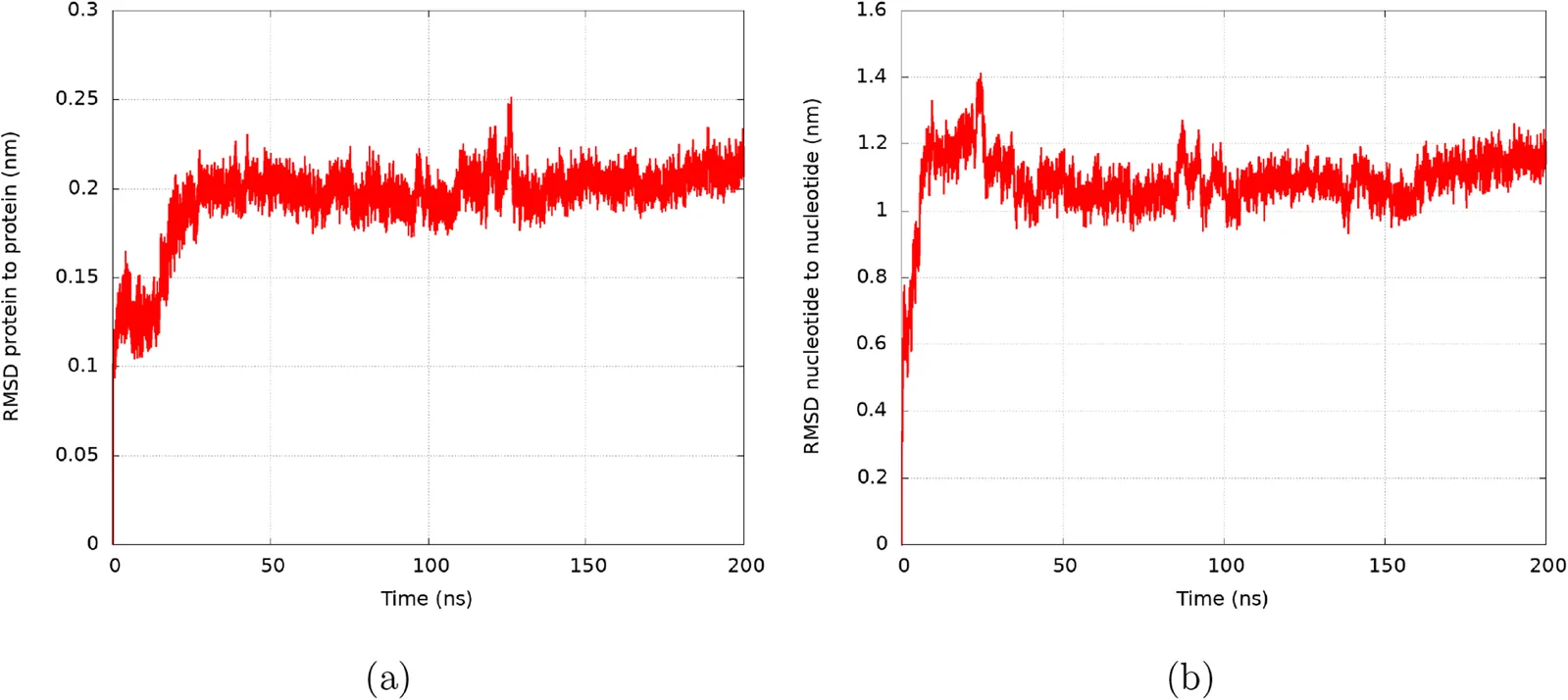
Root-mean-square deviation (RMSD) from the starting configuration of (a) protein with itself and (b) aptamer with itself, as a function of time. The protein reaches equilibrium soon after about 20 ns, while the aptamer needs 50 ns to reach equilibrium.

Next, let us analyze the root-mean-square fluctuation (RMSF) of the residues in equilibrium. Figure 4 shows these values for each residue of the protein and the aptamer. For our purpose, only the backbone atoms of the protein residues (the C_α_ atoms) and of the nucleotides (the C5′ atoms) are used for this calculation, thereby indicating the stability of the molecular backbones.

**Figure 4:**
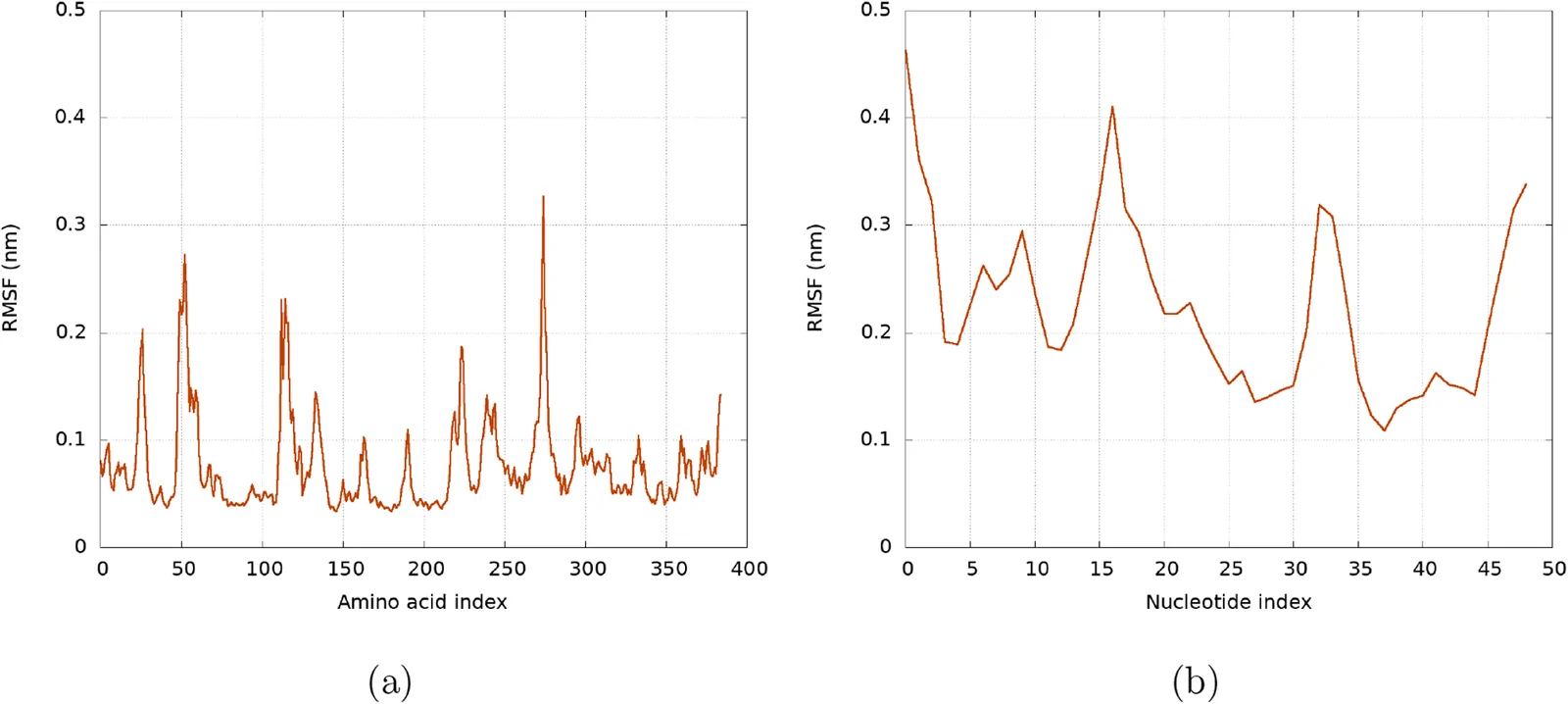
Root-mean-square fluctuation (RMSF) of each residue of (a) BoNT/A protein and (b) aptamer. Statistics are taken in equilibrium. The protein is very stable with little fluctuations. The aptamer part that binds the protein is also stable, while the tail-ends and the loops of the hairpins show flexibility.

Root-mean-square fluctuation (RMSF) is calculated as the time average of the deviation of atomic from its mean position. It shows highly mobile regions (like loops) versus rigid domains of the proteins. Typically, for a structurally stable protein or nucleic acid, RMSF values typically range from 1 to 2 Å for the core structural regions (helices, sheets, double helix), and about 3–5 Å for highly mobile regions such as terminal tails and exposed surface loops (see for example, ref 49 for nucleic acid simulation and ref 50 for protein simulation). Based on these criteria, it is evident that both the BoNT/A protein and the aptamer exhibit stability with all the core secondary structures have RMSF of less than 1 Å, and all the flexible loops have RMSF of less than 4 Å. Even the terminal regions of the aptamer demonstrate RMSF of less than 5 Å due to its elongated, extended single-strand conformation, which is not strongly tethered to the protein, still within acceptable stable value. Regarding regions with more flexibility, one notes that the nucleotides situated around the 16th–17th positions, located centrally within the first hairpin of the aptamer, also display relative flexibility. This observation can be readily comprehended as these nucleotides form the loop region of the hairpin, exposed to the solvent and do not engage significantly in protein binding, as evidenced in Figure 5. In fact, since the whole hairpin (8th–23rd positions) does not participate in the binding, the double-helix regions also exhibit higher RMSF than the double-helix regions of the second hairpin (26th–44th positions, represented by the red-colored tube in Figure 2). For both hairpins, the nucleotides within the central loop exhibit greater fluctuations. One may assert that the double-helix regions of the hairpins are important in facilitating BoNT binding.

**Figure 5:**
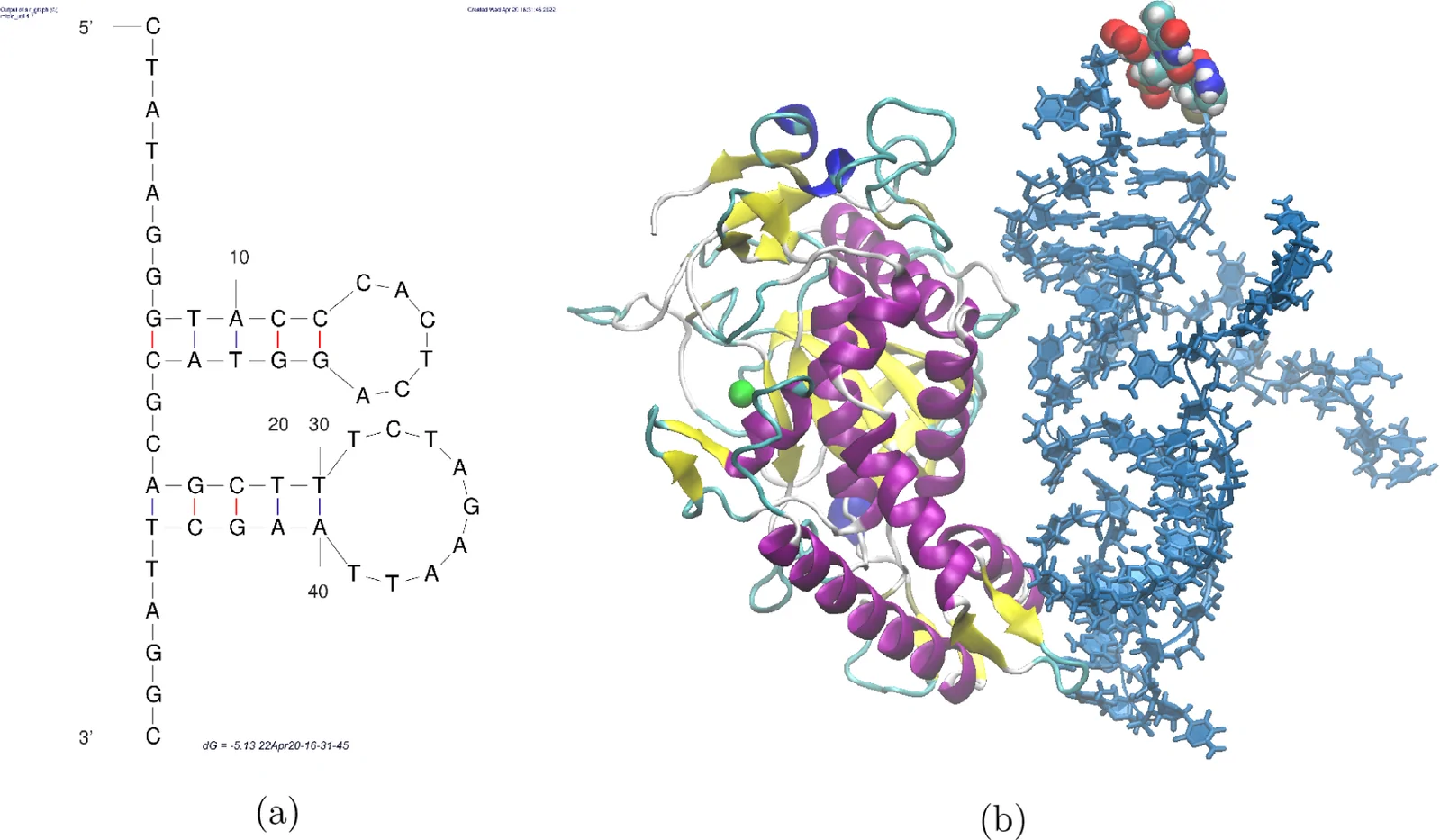
(a) The secondary structure of the aptamer A4 as predicted by mFold.^31^ It has two hairpins: the first hairpin is composed of nucleotides from positions 8th to 23rd, the second hairpin is composed of nucleotides from positions 26th to 44th. (b) The nucleotides 16th and 17th in the flexible loop of the first hairpin are shown as spheres. They have a larger amplitude of fluctuations in equilibrium because they do not bind strongly to the protein. The second hairpin is closer to the protein and has smaller fluctuations.

After investigating the structural stability of the complex, let us move on to analyze their interactions. To this end, let us calculate the amount of contacts between the molecules, specifically their coordination number. This quantity is defined using a smooth switching function to avoid abrupt variations:

1 $$S=\underset{i\in A}{\sum }\underset{j\in B}{\sum }\frac{1-(r_{ij}/r_{0}{)}^{n}}{1-(r_{ij}/r_{0}{)}^{m}}$$

where *r*_*ij*_ is the distance between atom *i* ^th^ of molecule A and atom *j* ^th^ of molecule B. The switching parameter, the cutoff distance *r*_0_, is set to 8 Å. The exponents *n* and *m* are set to the default values of 6 and 12, respectively. It is evident that, by this definition, at large separations between the atoms, the coordination number is 0, indicating an absence of contact, whereas at very small separations, *r*_*ij*_ → 0, the coordination number is 1, indicating a full contact. For practical computation of the coordination number, only one representative atom from each residue is selected. Specifically, similar to the RMSF calculation, the C_α_ atom from the amino acid and the C5′ atom from the nucleic acid are chosen as representative atoms of the respective residues.

In Figure 6, the coordination number between the BoNT/A protein and aptamer A4 is plotted as a function of time. It can be observed that the coordination number between the aptamer and the protein attains equilibrium approximately 50 ns into the simulation, which aligns well with the relaxation time of the aptamer (Figure 3b). The time of variation is within the relaxation time of the aptamer, suggesting that typical aptamer structural relaxation is related to its binding interface to the protein, not within the aptamer structure itself.

**Figure 6:**
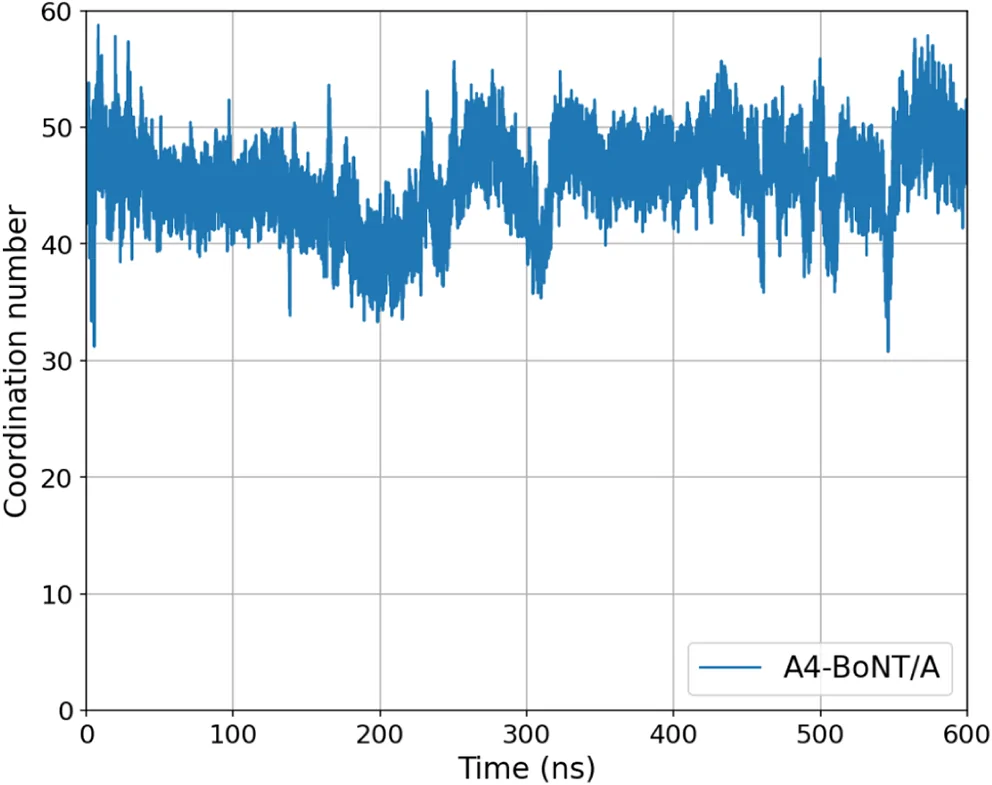
Coordination number between the residues of the aptamer A4 and the BoNT/A protein. This value is calculated using a smooth switching function with a cutoff *r*_0_ of 8 Å.

Lastly, the free energy of binding between the aptamer and the BoNT/A protein is computed. The solvent-implicit method of estimation MM-GBSA is used as stated in the Materials and Methods section. The result for A4 binding to BoNT serotype A is shown as a blue line in Figure 7. The characteristic time scale for the variation in free energy is approximately 50 ns, which is comparable to the aptamer’s aforementioned relaxation time. The average free energy of binding, calculated during equilibrium by excluding the initial 50 ns of the trajectory, is

2 $$\Delta G_{MM-GBSA}=-45.16\pm 11.34\mathrm{kcal}/\mathrm{mol}$$

**Figure 7:**
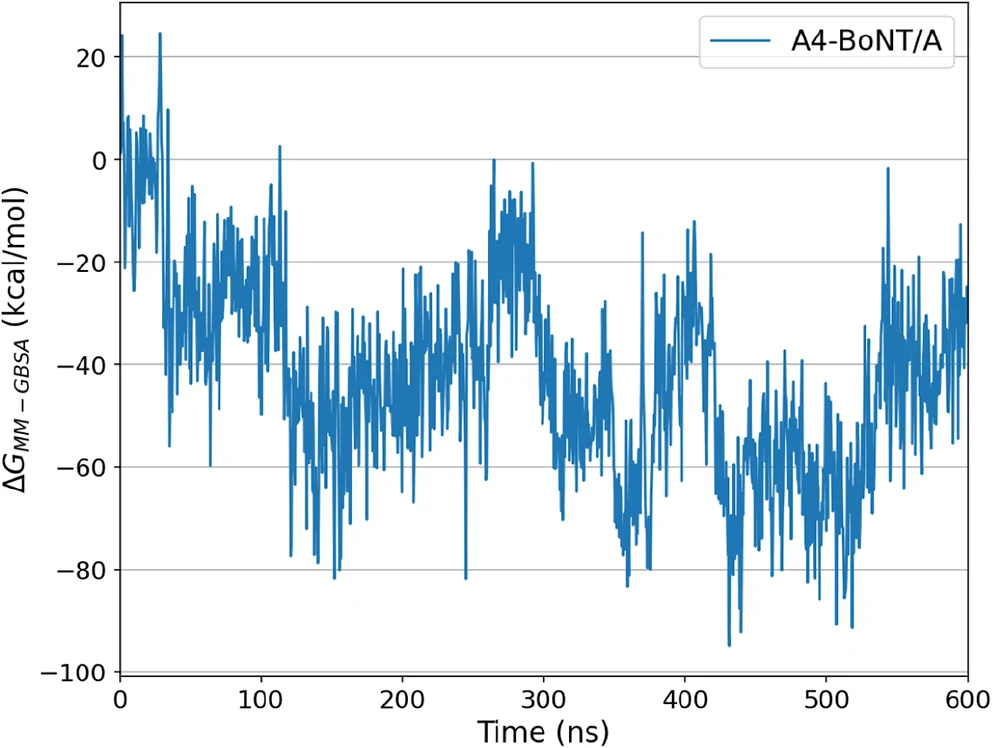
Free energy of binding, Δ*G*, of aptamer A4 to BoNT type A plotted as functions of time, calculated using the MM-GBSA estimation method.

Although the results exhibit considerable variability, they suggest that the relaxation of the system from the initial docking configuration results in a more favorable binding free energy. The equilibrium value of Δ*G* is less than that of the docking value of −30 kcal/mol. However, these figures should not be interpreted at face value since docking results are derived from a semiempirical formula. Nonetheless, the substantial free energy of binding is apparent. The values derived from molecular dynamics simulations are expected to be larger in magnitudes than those obtained from static docking analyses, as the latter neglects the relaxation of the complex that could further optimize its binding structure. This process cannot be captured in an almost static docking procedure.

### 3.3 Binding of an Aptamer to Three BoNT Serotypes

Having articulated the results and discussions concerning the typical structure and interactions associated with aptamer A4’s binding to its specific target protein, BoNT/A, let us investigate how this aptamer interacts with BoNT variants from other serotypes.

A particularly intriguing characteristic of the BoNT proteins is that even though they share only 30–35% sequence identity, their three-dimensional structures are very similar, as shown in Figure 8. In this figure, the top subfigure presents the outcomes of sequence alignments performed using the ClustalW Web server^51^ with the identical (*) and conserved (:) residues account for only 30% of the sequences. Conversely, the left subfigure, which displays the superimposition of the proteins, reveals a significant degree of structural similarity. Consequently, one might hypothesize that aptamers selected for binding to one serotype could also exhibit binding affinity to another serotype at analogous locations and, presumably, with comparable free energies of binding. The docking results displayed in Table 2 substantiate this hypothesis. All docking scores exceed −20 kcal/mol, indicating a robust potential for binding across all BoNT serotypes, as these scores are derived from known aptamers with established binding capabilities to BoNT. Furthermore, the docking outcomes suggest that the variability among the structures of the aptamers, particularly in their secondary structures (which can influence the exact binding position), has a more pronounced effect on the binding free energy than the variations observed among the serotypes. This provides justifications for experimentalists’ pursuits of cross-type botulinum neurotoxin detection.^25–28^

**Figure 8:**
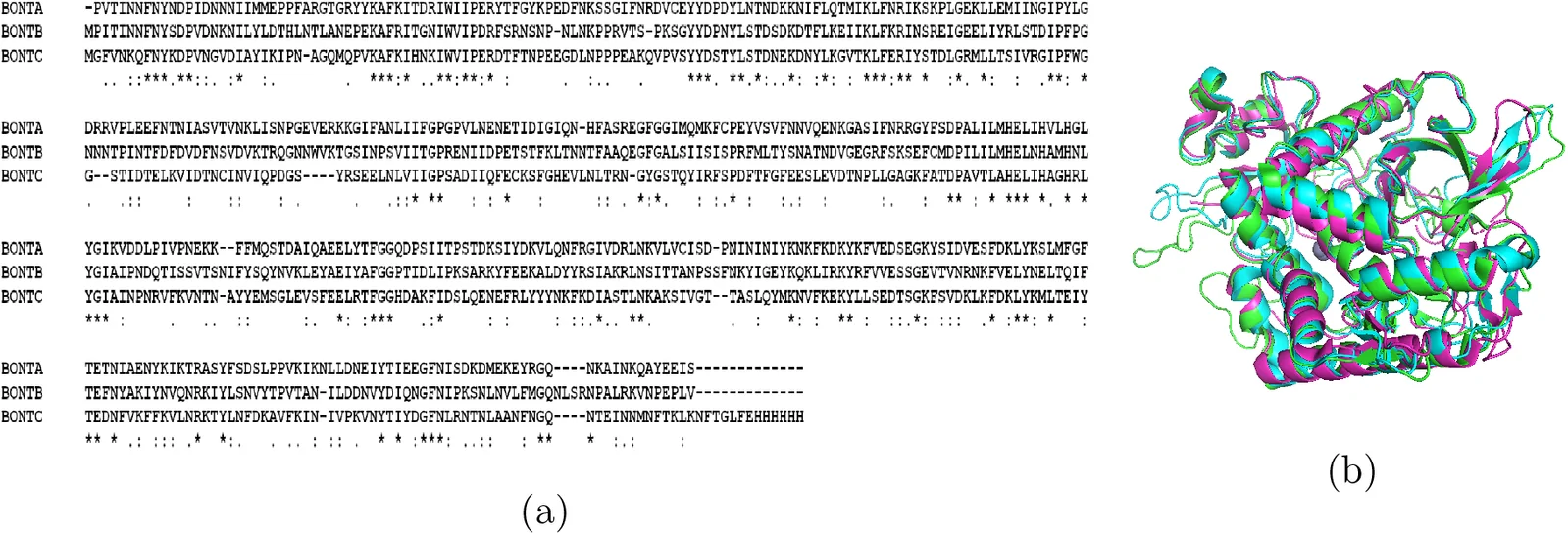
(a) The sequence alignments of the three botulinum neurotoxin serotypes A, B, and C, showing only about 30–35% sequence identity among them. Below the protein sequences is a key denoting conserved sequence (*), conservative mutations (:), semiconservative mutations (.), and nonconservative mutations (“blank″); (b) the three-dimensional structures of all three BoNT serotypes are very similar, especially for the secondary structures.

Let us examine how this variability among serotypes manifests in the analysis of molecular dynamics simulations. To further elaborate on the preceding subsection, in this section, we shall present results specifically pertaining to aptamer A4. In Figure 9a, the free energy of binding, Δ*G*, of aptamer A4 to BoNT types A, B, and C is plotted as a function of times. It is evident that these Δ*G* values appear to converge toward similar magnitudes (albeit within considerable fluctuations) among the three BoNT proteins as equilibrium is attained. Overall, the value Δ*G* is smaller than that of the docking value, indicating further relaxation in the molecular dynamics simulation from the initial docking configuration. Aptamer A4 binds well to all three BoNT serotypes, with a binding free energy of approximately −45 kcal/mol.

**Figure 9:**
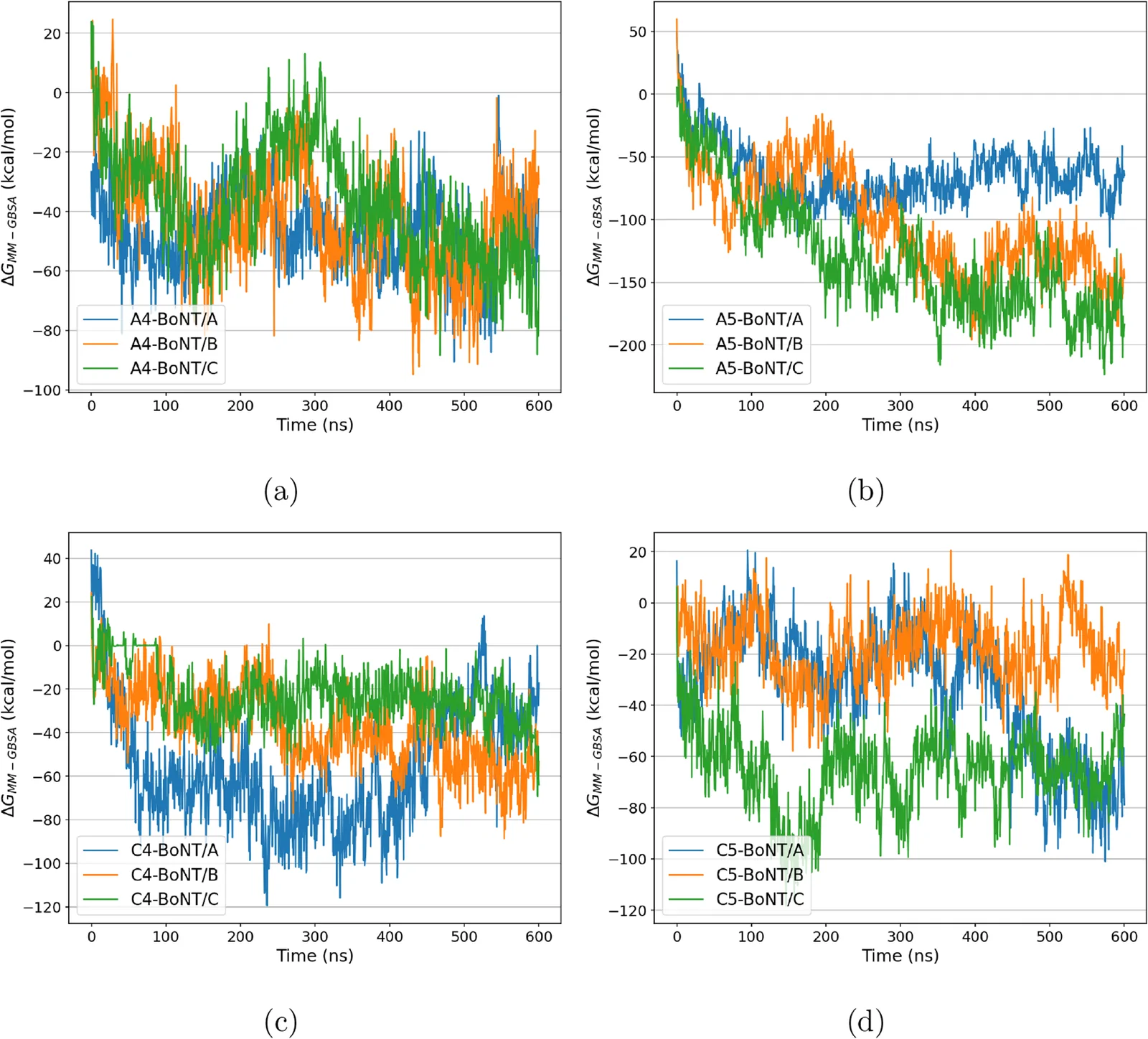
Free energy of binding, Δ*G*, of aptamers A4 (a), A5 (b), C4 (c), and C5 (d) to BoNT types A, B, and C plotted as functions of time. A5 aptamer seems to show the highest binding energy to all three serotypes.

### 3.4 Similarities and Differences among the Aptamers

Having conducted an extensive analysis of aptamer A4 in its interactions with botulinum neurotoxin (BoNT) across various serotypes, we are now equipped to elucidate the selectivity of the engineered aptamers concerning BoNT proteins along with their respective similarities and divergences. Let us start with the examination of the binding free energies between the aptamers and the BoNT proteins of serotypes A, B, and C. Figure 9 shows the binding free energies as functions of time for the complexes of aptamers A4, A5, C4, and C5 with the BoNT of different serotypes. One sees that A4 and A5 bind well to all three serotypes. As stated in the previous section, the A4 aptamer binds to all BoNTs with almost the same strength of −45 kcal/mol. Aptamer A5 also exhibits robust binding energies toward all three serotypes, potentially exceeding that of the A4 aptamer. However, it also displays a broader range of fluctuations. In the case of aptamer C5, it distinctly favors binding to its specific target, BoNT/C, while exhibiting significantly reduced binding affinity toward BoNT/A and BoNT/B. Consequently, if we choose to engineer biosensors only targeting BoNT/C, aptamer C5 is the most promising option, but for cross-binding, A4 and A5 would be better options.

A notable characteristic shared among the three aptamers is that the secondary structures of A4, A5, and C5 each comprise two hairpin formations, one of which includes double helices consisting of 5–6 nucleotide base pairs (Figure 10). The double-helix part of the hairpin is almost continuous, without any mismatching in the middle. In molecular dynamics, this helix part is stable and rigid with small RMSF values. The docking pose shown in Figure 2 shows that one of these short and rigid helices binds directly to the BoNT, while the other double helix engages in a reversed direction. Although this structural aspect is limited to our small set of aptamers, it is interesting to note that, in ref 33, 24 different ssDNA sequences with experimentally resolved aptamer hairpin structures also show helices with 5–6 nucleotides. It is indeed advantageous to investigate the prevalence of this motif as additional structural data become available through experimental methods.

**Figure 10:**
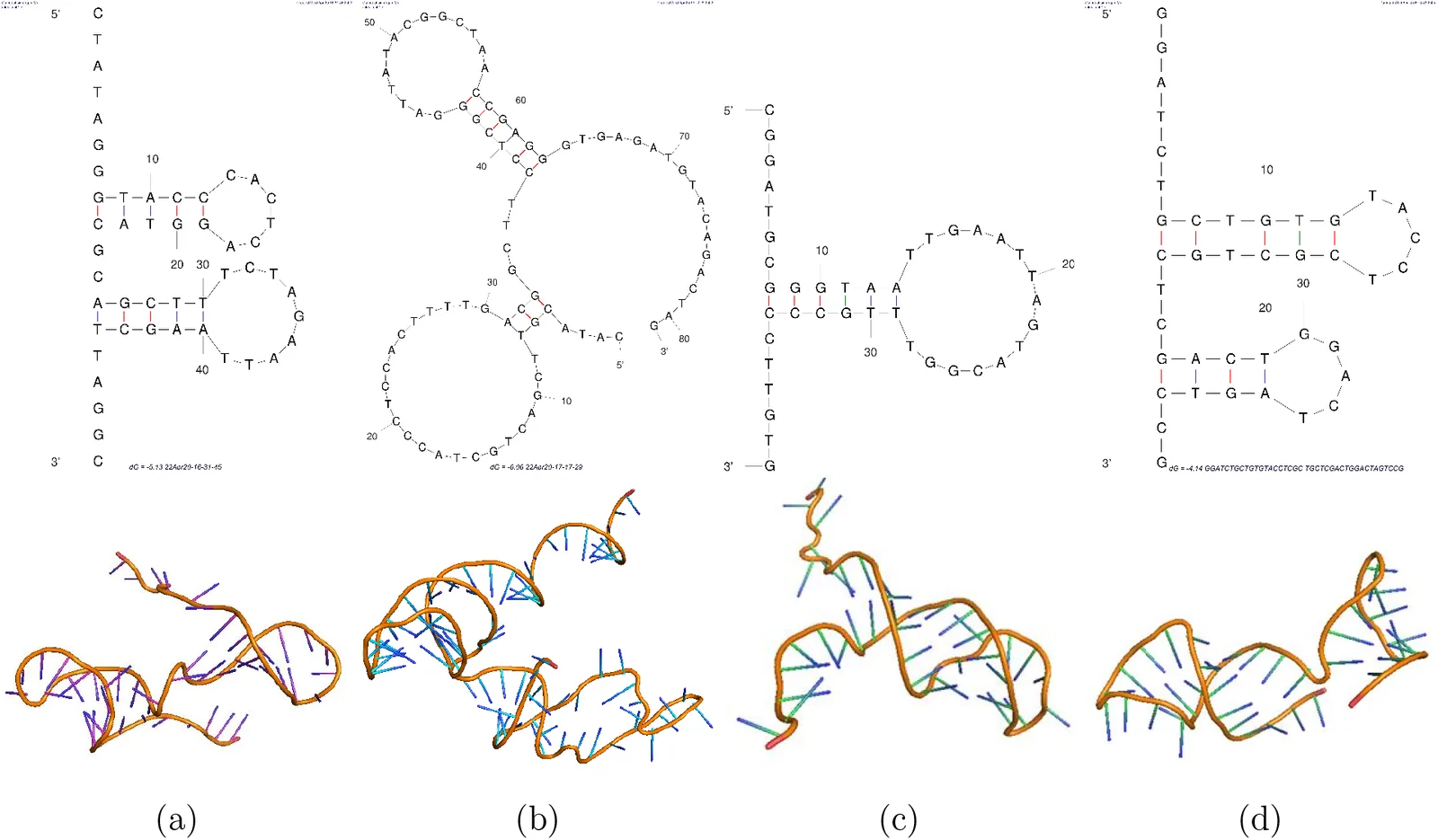
Top row: the secondary structure of A4 (a), A5 (b), C4 (c), and C5 (d) aptamers obtained from the mFold program. The bottom row is the three-dimensional structures of the aptamers constructed as described in the Materials and methods section. The three most promising aptamers (A4, A5, C5) all contain two hairpins with the rigid double-helix part composed of 5–6 nucleotide base pairs continuously without any mispairing in the middle.

Figure 9 shows that the aptamer C4 has the least favorable binding free energy as compared to A4, A5, and C5. It has only one hairpin with a very long flexible loop in the central region. Similar secondary structure analyses (not shown) for other aptamers investigated in this work show that they do have a hairpin structure, but either their double-helix part is too long, or not continuous, or the hairpin has a large loop section. This observation appears to contribute to a diminished docking score, or a less favorable Δ*G* after molecular dynamics simulation.

Lastly, for concreteness, we list all the binding free energy of the four aptamers undergone molecular dynamics simulation in Table 3. It agrees with the qualitative discussions we already mentioned above. All aptamers bind to the BoNT protein with good binding free energy of −20 kcal/mol or greater. For BoNT/C binding, A5 and C5 are the most promising candidates. However, for cross-type detections, A4 and A5 demonstrate superior characteristics. A5 binds stronger with the strength of −75 kcal/mol, but has larger uncertainty and variation, while A4 binds less with the strength of −45 kcal/mol, but it is much more uniform, with less fluctuations.

**Table 3:** Binding Free Energy of the Aptamers with the BoNT Proteins as Obtained from Molecular Dynamic Simulations^a^

Aptamer	BoNT/A	BoNT/B	BoNT/C
A4	–46.70 ± 12.92	–46.57 ± 17.09	–39.59 ± 18.89
A5	–71.30 ± 15.65	–109.76 ± 39.14	–145.21 ± 31.88
C4	–59.23 ± 24.33	–41.12 ± 16.95	–26.65 ± 10.44
C5	–36.31 ± 24.67	–18.87 ± 13.31	–68.33 ± 14.48

a All values are in the unit of kcal/mol.

These quantitative distinctions were not apparent during the docking procedure, underscoring the importance of structural relaxation and molecular dynamics exploration in aptamer-protein systems.

It should be stressed that this work relies heavily on the MM/GBSA method to provide approximate relative binding energies, and entropic contributions are neglected. Experimental binding affinity measurements would serve as a necessary means to substantiate our findings; however, it is currently beyond the scope of our manuscript. Nevertheless, the selected aptamers were chosen from experimental studies that included binding affinity tests using SPR and nitrocellulose filter retention assays, providing valuable baseline reference data. The dissociation constants of the BoNT/C aptamers ranged from 42.8 nM to 0.689 nM. The reported sandwich ELISA confirmed the selectivity of these aptamers against BoNT/E and BSA. The *K*_D_ values for BoNT/A aptamers were in the nanomolar range (87–186 nM), being specifically bound to the target when tested with other His-tagged proteins, streptavidin, and lysozyme. Although the binding free energies calculated from MD results are stronger than those experimentally indicated binding affinities of tens of nanomolar to subnanomolar range, comparison among the MD results suggests that measurements would give similar binding affinities when cross-serotype detections are performed for our candidates.

## 4 Conclusion

The comprehensive computational work by using molecular docking and molecular dynamics simulation carried out in this paper presents a step toward the goal of better experimental design of biosensors for cross-species detection of botulinum neurotoxin types A, B, and C, which are the aims of several experimental groups. We selected to explore established aptamers targeting BoNT/A and BoNT/C to ascertain their potential for cross-binding to other serotypes. In this way, we can compare their structural configurations and interaction profiles, facilitating the identification of similarities and disparities that elucidate the fundamental physical mechanisms at play. Computational investigations can provide insights and direction toward improved design of aptamer structures and better experimental aptamer selection and/or synthesis, saving time, labor, and material costs.

Given that aptamers are large biomolecules with numerous degrees of freedom, conventional docking methodologies that rely on static structures are inadequate for capturing the significant structural relaxation of the protein–aptamer complex upon binding. Our findings distinctly emphasize the necessity of molecular dynamics simulation for any rigorous computational inquiry into binding structures and interactions.

During the simulation, the proteins (BoNT/A, BoNT/B, or BoNT/C) deviate minimally from their starting configuration during MD simulations, as low as 1.5 Å RMSD. The aptamers do not compromise the integrity of the protein but rather contribute to its enhanced stability. Consequently, this allows these aptamers to be good candidates for biosensors for detection. The aptamer shows stronger relaxation, with RMSD ranging from 2 to 4 Å. Importantly, the coordination number between the aptamers and the proteins strongly correlates with the binding free energy, suggesting that most of the structural relationships of the aptamers are related to their binding interfaces with the proteins.

Docking results show that all of the aptamers show good binding affinity to three types of aptamers. This seems to stem from the fact that although less than 35% sequence identity, all proteins have similar three-dimensional structures. Aptamers that bind well to one serotype should bind to the other serotypes at similar locations, with similar free energies. The variation in binding free energies between different aptamers is higher than the variation in binding energies of different serotypes. The four most promising aptamers from docking A4, A5, C4, and C5 are chosen for molecular dynamics simulation. A4 and A5 remain binding to all three BoNT serotypes with similar binding free energy within uncertainty, while C5 clearly demonstrates stronger affinity toward its designated target of BoNT/C. C4 displays inferior performance among the aptamers.

For cross-type detections, aptamers A4 and A5 are promising candidates. A5 binds stronger with the strength of −75 kcal/mol, but has larger uncertainty and variation. A4 binds slightly less with the strength of −45 kcal/mol but it is much more uniform among the serotypes.

For all aptamers investigated, there are two binding sites for an aptamer on BoNT, which are located on opposite sides of the protein. This suggests the experimental setup of a sandwich array to allow aptamers to bind to both binding sites for optimal sensitivities, which was already implemented for BoNT/C ref 29.

In this work, rather than designing from the beginning, we select aptamers from those that are already verified experimentally for individual BoNT serotypes and investigate their molecular mechanism of binding. We examined their molecular binding mechanisms and investigated the potential for utilizing these aptamers in cross-type detection using computational approaches. In the near future, we plan to expand this work further to experimentally validate our computational results as well as possibly design better aptamer sequences based on the knowledge obtained from this molecular study.

Overall, our computational results provide important insights into the molecular binding mechanism, support current experiments, and assist in further optimization and design of new aptamers for these botulinum neurotoxin proteins, as well as for a broader spectrum of clostridial neurotoxins in general.

## Data Availability

All the MD trajectories generated and analyzed in the current study are available from the corresponding author on reasonable request.
